# Laetoli’s lost tracks: 3D generated mean shape and missing footprints

**DOI:** 10.1038/srep21916

**Published:** 2016-02-23

**Authors:** M. R. Bennett, S. C. Reynolds, S. A. Morse, M. Budka

**Affiliations:** 1 Institute for Studies in Landscape and Human Evolution, Faculty of Science and Technology, Bournemouth University, Fern Barrow, Poole, BH12 5BB, UK

## Abstract

The Laetoli site (Tanzania) contains the oldest known hominin footprints, and their interpretation remains open to debate, despite over 35 years of research. The two hominin trackways present are parallel to one another, one of which is a composite formed by at least two individuals walking in single file. Most researchers have focused on the single, clearly discernible G1 trackway while the G2/3 trackway has been largely dismissed due to its composite nature. Here we report the use of a new technique that allows us to decouple the G2 and G3 tracks for the first time. In so doing we are able to quantify the mean footprint topology of the G3 trackway and render it useable for subsequent data analyses. By restoring the effectively ‘lost’ G3 track, we have doubled the available data on some of the rarest traces directly associated with our Pliocene ancestors.

The Laetoli site (Tanzania) contains the oldest known hominin footprints, dated to 3.66 Ma^1^. Since their discovery in 1978, they have been subject to many thousands of words, as authors have argued and analysed their significance in terms of the evolution of hominin foot morphology, function and gait^2^,^3^,^4^. The two hominin trackways were probably made by *Australopithecus afarensis* and are preserved in volcanic ash. They represent at least three individuals in two parallel trackways, with one being a composite formed by at least two individuals that walked in single file (Fig. 1a). Most researchers have focused on the single, clearly discernible G1 trackway (Fig. 1a,b) and for over 35 years the G2/3 trackway (Figs 2 and 3) has been largely dismissed as a less-informative composite and therefore unable to contribute data to anatomical and biomechanical analyses^5^.

Recent advances in vertebrate ichnology^6^,^7^,^8^ have been facilitated by the increased availability and use of three-dimensional digital data and have placed the analysis of hominin tracks on a more objective footing. For example, such data has allowed geomorphometric techniques and whole-foot track registration methods to be applied. As a result more sophisticated statistical analyses can be performed and in some cases, mean tracks have been computed for a given trackway or population of tracks^9^,^10^,^11^. We have developed a method of track registration which is ideally suited for extracting and comparing complex tracks that uses landmarks to match similar points on multiple tracks. We have applied this approach to the G2/3 Trail in order to extract the detailed topology of the uppermost (G3) track from the composite, to create a mean track from the available data (N = 5; Fig. 1c).

## Track registration and methods

Direct comparison of tracks, either between subjects or within subjects, requires the registration^12^ of one or more tracks, such that areas of anatomical similarity are optimally overlapped as defined by the user or by some form of statistical parameter (e.g., least squares). Registration refers to the process of transforming one track, termed a ‘template’ (or here ‘registered’) to a ‘source’ (or here ‘master’) track, such that the three-dimensional topology of each track is optimally overlapped. Once a succession of tracks have been registered, it is possible to compare the ‘z’ values (i.e., depth) for each track, by using some form of sampling grid over the registered tracks, and thereby compute measures of central tendency for the population of registered tracks. A mean, or median, track created in this way provides, in theory at least, a more accurate topological representation of the track-maker’s foot impression than any one individual track. A mean track is free from intra-track variability within in a trackway caused, for example, by variation in sedimentary properties along a trackway, or step-specific gait occurrences due to occasional foot slips, for example. It therefore draws out the recurring topological (i.e. depth variation) track morphology, which by inference, gives insight into the biomechanical signature left by the track-maker’s mode of gait. Of course, as with any mean, this depends on the size of the sample and the degree of variance within the sampled population. Despite this caveat, this method provides a robust way of statistically comparing different track populations.

Using an approach drawn from neuroimaging, Pataky and Goulermas developed a registration method for plantar pressure records, based on statistical parametric mapping (SPM), which they term pedobarographic Statistical Parametric Mapping (pSPM)^10^. Registrations were achieved by various automated algorithms using a progressive approach in which tracks are registered first to an initial track (the first in the series) and then re-registered to initial computed mean. In subsequent work, Pataky and colleagues compared different registration methods^13^, finding that most global approaches (e.g., mean squared error, probability weighted variance) performed in a similar fashion and better than reduction-based methods (e.g., principal axis). Significantly, manual methods were as comparable to automated ones when averaged between operators. A pSPM approach has been applied to footprint studies by substituting pressure for depth^14^,^15^. This approach is not without its limitations, however. The chief issue is that for automated registration the topology of the tracks needs to be smooth and relatively similar across a range of tracks. However, fossil tracks contain marginal forms, which vary between tracks, and these may interfere with automated registration. One has to, therefore, either remove such distractions by cropping a track vertically through the application of a vertical height threshold to focus solely on the plantar surface or, alternatively, use a manual registration approach. The manual registration tools in pSPM are currently limited and based solely on overlapping two print outlines and the pSPM code which runs in MATLAB is not particularly user-friendly or widely available at present. In order to overcome these issues and, more importantly to create a system designed specifically to deal with footprints rather than pressure records, the authors have independently developed an alternative software-based analytical solution.

This software solution, called Track Transformer, is written in Python and facilitates a complete track processing workflow, from loading raw input files in a variety of formats, performing basic measurements, via registration of a collection of prints through landmark matching, to producing a set of statistics describing the registered tracks (Supplementary Information, Fig. S1). The registration process requires the user to denote one of the prints as the ‘master’ with which all the remaining prints are aligned, and to define a set of corresponding landmarks (effectively matching points) for each track. Selection of the master is normally guided by identifying the track that is most topologically complete. Landmarks can be placed on the basis of either formally anatomically-defined points, or more commonly informally based on points of recurrence (i.e. matching the same topological point on the two tracks). These landmarks can also be complemented by “geometrical” landmarks, located, for example, between defined landmarks. Track Transformer currently supports three types of geometrical landmarks: line, triangle and square, where an artificial landmark is inserted in the centre of gravity of each pair, triplet or quadruplet of the user-defined landmarks, respectively. The aim here is to optimise the registration. The software then computes a transformation of the registered track to align it with the master, by minimising the mean squared deviation between the landmark coordinates in the xy-plane. If we denote 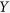 as a matrix of landmark coordinates for the master print (one landmark per row) and by 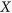 a matrix of corresponding landmark coordinates of the print to be registered, the software calculates the transformation matrix 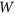 as an approximate, optimal in the least square sense, solution to the following system of equations:


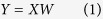


As with pSPM^10^ Track Transformer supports two types of transformations. Firstly, affine transformation, for between-subject registrations, where the matrix 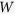 is not constrained in any way and is calculated as:


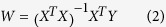


where the matrix 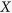 is supplemented by a column of 1′s to account for the intercept term, hence allowing for translation.

Secondly, rigid transformation, for within-subject registrations, where the matrix 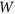 is constrained to represent a valid rotation only. Denoted by *A* = *X*_*C*_^*T*^*Y*_*C*_ the covariance matrix of 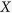 and 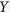 after centring (i.e. subtracting their respective centroids, which accounts for translation), the optimal transformation can be calculated as:


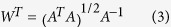


These transformations are implemented for numerical stability using the Kabsch algorithm^16^, which calculates 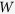 via singular value decomposition of the covariance matrix. Once tracks are registered the software then samples the stacked or registered tracks at various resolutions (0.25, 0.5 and 1.0 mm) to compute a frequency distribution of values for each point from which measures of central tendency can be computed and displayed visually (i.e. display a mean or median track).

The Laetoli track data are based on optical laser scans (Konica-Minolta VI900) of first generation casts held at the National Museums of Kenya (Nairobi) and were collected with kind permission from the curatorial staff by the senior author in 2008. All registrations were undertaken by multiple operators using self-chosen landmarks (Supplementary Information, Fig. S2). A mean track for the G1 trail was calculated using the following eleven tracks: G1-23, G1-25, G1-26, G1-27, G1-31, G1-33, G1-34, G1-35, G1-36, G1-37 and G1-39. The Daasanach mean used as a modern comparator is based on tracks from 33 different individuals living close to Ileret (northern Kenya). Tracks were selected from a sequence of half a dozen in an experimental tray filled with fine sand and silt taken from excavated spoil at FwJj14E site in 2008^17^. The tracks were scanned with a Konica-Minolta VI900 optical laser scanner.

**Figure 1:**
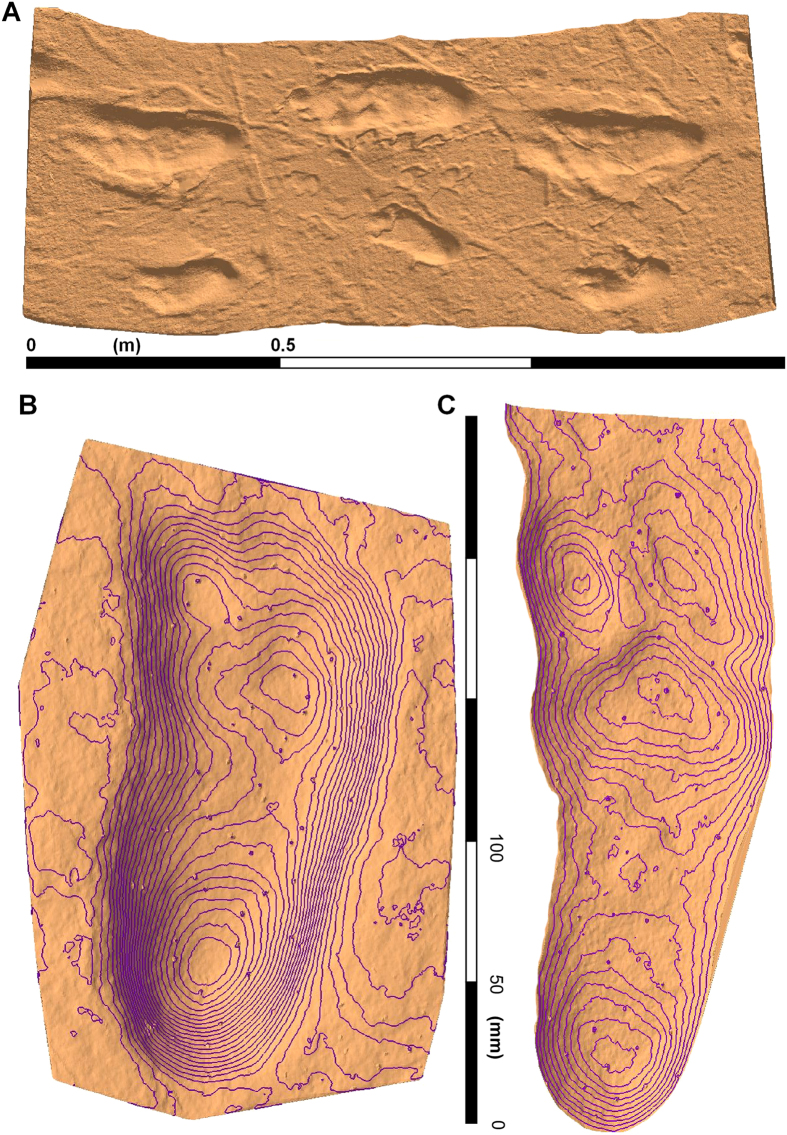
(**A)** Colour-rendered optical laser scan of part of the Laetoli trackway showing both the G1 and G2-3 composite trails. (**B)** A mean track for the G1 trail computed from eleven individual tracks, the contour interval is 1 mm. (**C**) A mean track for the G3 trail computed from five individual tracks, the contour interval is 1 mm. The data was captured using a Konica-Minolta Vi-900 scanner, processed in Foot Processor (http://footprints.bournemouth.ac.uk/) and ArcGIS Version 10 (http://www.esri.com/software/arcgis).

Tracks from the G2 and G3 trackways were imported into Track Transformer and registration was based on the visible G3 tracks and was repeated with different combinations of tracks and with different ‘masters’, as defined by different operators (Supplementary Information, Fig. S3). The registered G3 Track mean shown in Fig. 1 is based on: G2-18, G2-26, G2-27, G2-28 and G2-29. Once a satisfactory registration for the G3 tracks was obtained, a mean was computed and a vertical elevation threshold applied to remove peripheral noise not pertaining to the track. Mean tracks were compared in Track Transformer and registered tracks saved so that they could be imported into the GIS software package ArcMap as CSV files and contoured for enhanced presentation (Fig. 4). A mean for the whole of the G2/3 composite tracks was not calculated since the overlap between the two track-makers was not consistent and therefore such a mean does not represent a recurrent pattern.

## Results

**Figure 2:**
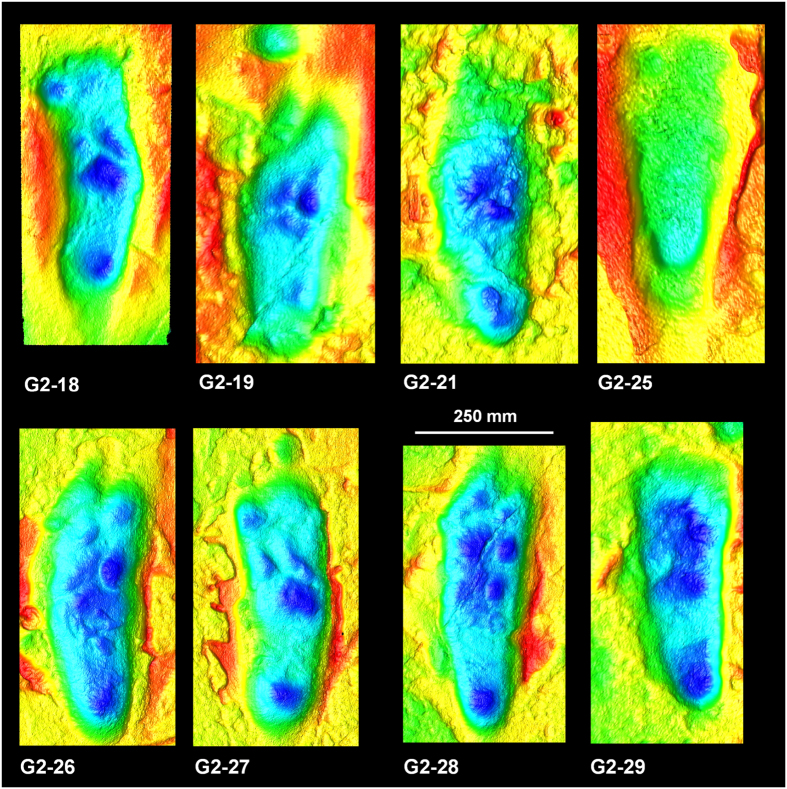
Colour-rendered optical laser scans of a selection of tracks from the G2 Trail at Laetoli. Warm colours denote high elevation, and cool tones indicate low elevations. Note the three potential hallux impressions in G2-28 which may be indicative of three rather than two track-makers, as is commonly inferred. The curved ‘banana-like’ form of G2-18 is typical of the whole track and suggests that the G3 track cut the heel of the earlier G2 track. The same is true of G2-27. The data was captured using a Konica-Minolta Vi-900 by the authors and processed in Polygon Editing Tool Version 2.4 (http://www.konicaminolta.com/instruments/index.html).

The extracted G3 track mean is shown in Fig. 1C and compared to the G1 and Daasanach means in Fig. 4. The G3 mean is slightly longer than the G1 mean (G1 193 mm, G3 228 mm; Heel to second toe). Using the empirical relationship of Tuttle and colleagues^18^ this gives a difference in estimated height between the two track-makers of 0.23 m (G1 1.3 m, G3 1.53 m), which is marginally higher than reported by Tuttle and colleagues (G1 1.26 m, G3 1.41 m). In terms of track topology, both show an abducted hallux, while the lesser toes are more clearly defined in the G3 than in the G1 mean (Figs 1a,b and 2b). The heel depth of the G3 is less pronounced than that of the G1 and the contour outline within the heel base more semi-circular which contrasts with the oval shape aligned along the long axis of the track in the G1. The plan-form geometry of the forefoot is also different, being more elongated along the sagittal plane in the case of the G3 mean. Forefoot and heel depths are similar in the case of the G3 mean, whereas heel depths are greater in the case of G1. The comparative anatomy of the G1 to the Daasanach means reveal key differences in the shape and depth of the heel; the degree of hallux abduction; and the definition of the lesser toes (Fig. 4b). Between the G3 and Daasanach tracks (Fig. 4b) the key differences are: the degree of hallux abduction, the lack of lateral depth in the mid-foot region in the G3, and the degree of medial weight transfer in the forefoot region which is greater in the modern track.

We propose three possible explanations for the observed differences between the G1 and G3 track means. Firstly, it is just conceivable that the G1 and G3 trails could have been made by different hominin species, although *Australopithecus afarensis* is the only hominin species recognised from Laetoli^19^. It is worth noting that some authors have argued that the trackways are the result of a hominin more anatomically modern than *Australopithecus afarensis*^20^. In the absence, however, of any other evidence and on the grounds of parsimony, we reject this explanation. Secondly, the differences may be accounted for by substrate and the compressed nature of the composite G2/3. In firmer substrates only those areas receiving maximum plantar pressure leave an impression^15^,^21^,^22^ and in the case of the G3 only those areas receiving the maximum plantar pressure may be recorded. Finally, the differences may be explained by individual variation in foot anatomy/size and sexual dimorphism.

There has been extensive debate as to whether the G1 Trail was made by a track-maker with: a foot that is essentially modern both in terms of anatomy and biomechanical function such that the tracks are indistinguishable from those made by the foot of a habitually unshod *Homo sapiens*; or by a foot that is essentially distinct, or at least intermediate in form, from that of *Homo sapiens*^23^. In a recent analysis^14^ using a whole-foot comparison of a G1 mean track with modern tracks it is argued that the G1 track were made by a hominin with an essentially modern biomechanical foot function. The G3 track supports this conclusion, and may strengthen it further, since the G3 mean track is strikingly similar to a modern track (Figs 1c and 4b). Note the similar heel geometry, hallux depth and the presence of forefoot rotation over the metatarsal heads. There is, however, a slightly less pronounced medial transfer of mass in the forefoot area, perhaps indicative of some difference in foot efficiency in the latter stages of stance. Importantly, this study means that we now have two mean tracks available, made by two different individuals with which to explore and debate these questions further.

**Figure 3:**
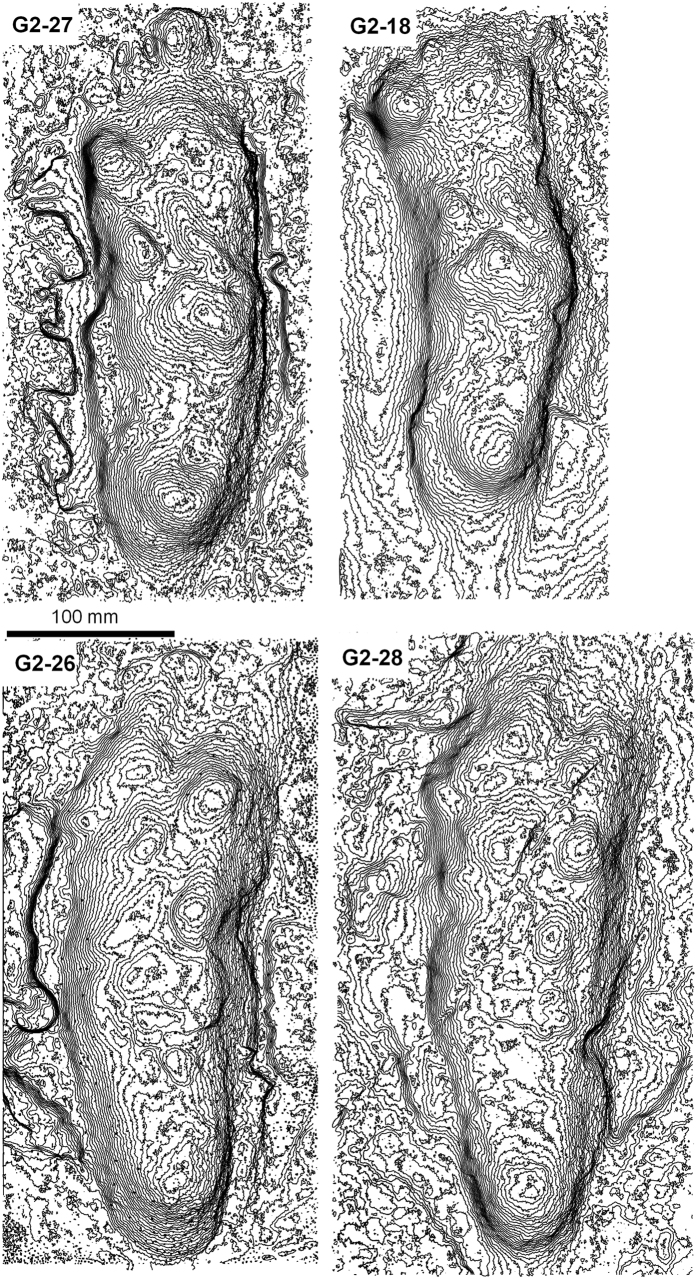
Contour maps for a selection of tracks from the G2 Trail at Laetoli. Contour interval is 1 mm. Note how distinctive the G3 tracks are in all four of these examples. Also note the slight hint in track G2-28 of a third unknown print. The data was captured using a Konica-Minolta Vi-900 scanner, processed in Foot Processor (http://footprints.bournemouth.ac.uk/) and ArcGIS Version 10 (http://www.esri.com/software/arcgis).

**Figure 4:**
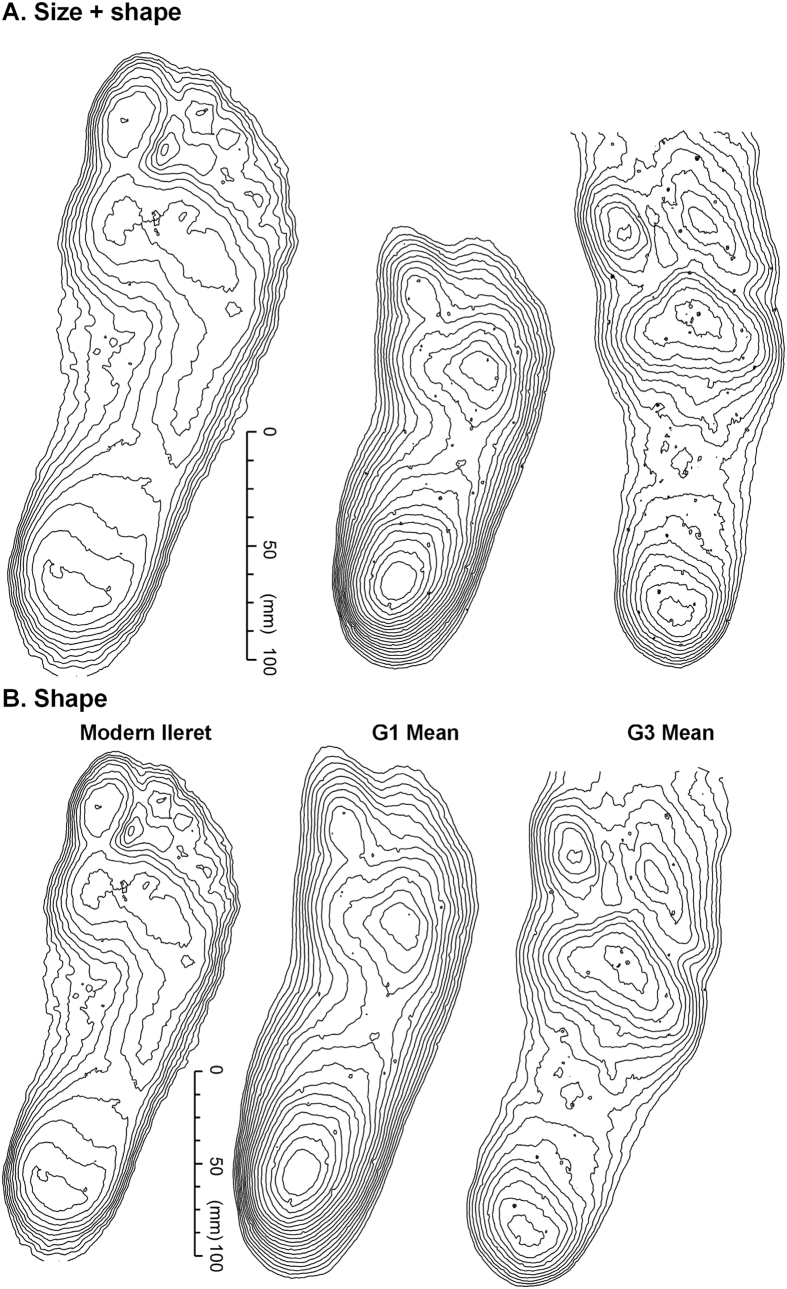
(**A**) Contour maps (1 mm interval) for unregistered mean tracks for habitually unshod modern feet (Daasanach, northern Kenya N = 33), G1 Trail (N = 11) and G3 Trail (N = 5). (**B**). Contoured maps for registered mean tracks for habitually unshod modern feet (Daasanach, N = 33), G1 Trail (N = 11) and G3 Trail (N = 5). The data was captured using a Konica-Minolta Vi-900 scanner, processed in Foot Processor and Track Transformer (http://footprints.bournemouth.ac.uk/) and ArcGIS Version 10 (http://www.esri.com/software/arcgis).

It is worth noting that the multiple track-makers of the G2/3 trackway did not follow exactly in one another’s tracks, as is commonly asserted. Footfall was offset with the G3 track-maker frequently cutting through the heel of the earlier G2 track (see: G2-18, G2-27, Figs 2 and 3). It is also worth noting that in one specific print (G2-28) there is potential evidence to suggest that three track-makers may have been involved, as evidenced by the presence of three potential hallux impressions (Figs 2 and 3). It is therefore possible that additional track-makers may have been involved.

In conclusion, the application of a new whole-foot technique has allowed the “lost” G3 track to be visualised accurately for the first time, effectively doubling the data available for biomechanical and anatomical debate. It is for the community to consider the implications of this additional data to the ongoing debate about early hominin locomotion and its role in human evolution. We also draw attention to the potential of track registration methods, like that illustrated here, for use in wider studies of vertebrate ichnology, including the study of dinosaur tracks.

## Additional Information

**How to cite this article**: Bennett, M. R. *et al.* Laetoli’s lost tracks: 3D generated mean shape and missing footprints. *Sci. Rep.* **6**, 21916; doi: 10.1038/srep21916 (2016).

## Supplementary Material

Supplementary Information
