# Supplementary material for: Laetoli’s lost tracks: 3D generated mean shape and missing footprints
Source: Sci Rep. 2016 Feb 23;6:21916. doi: 10.1038/srep21916 (PMC4763286; doi:10.1038/srep21916)
Supplement: Supplementary Information [file srep21916-s1.pdf]

Supplementary Information

Laetoli’s lost tracks: 3D generated mean shape and missing footprints

Bennett M.R., Reynolds S.C., Morse S.A., Budka M.

Institute for Studies in Landscape and Human Evolution, Faculty of Science and Technology, Bournemouth University, Fern Barrow, Poole, BH12 5BB, UK.

This supplementary information document contains the following data:

1. Additional Figures S1 to S5; this document.
2. Data (xyz coordinates) for the G3 mean track; separately data file.

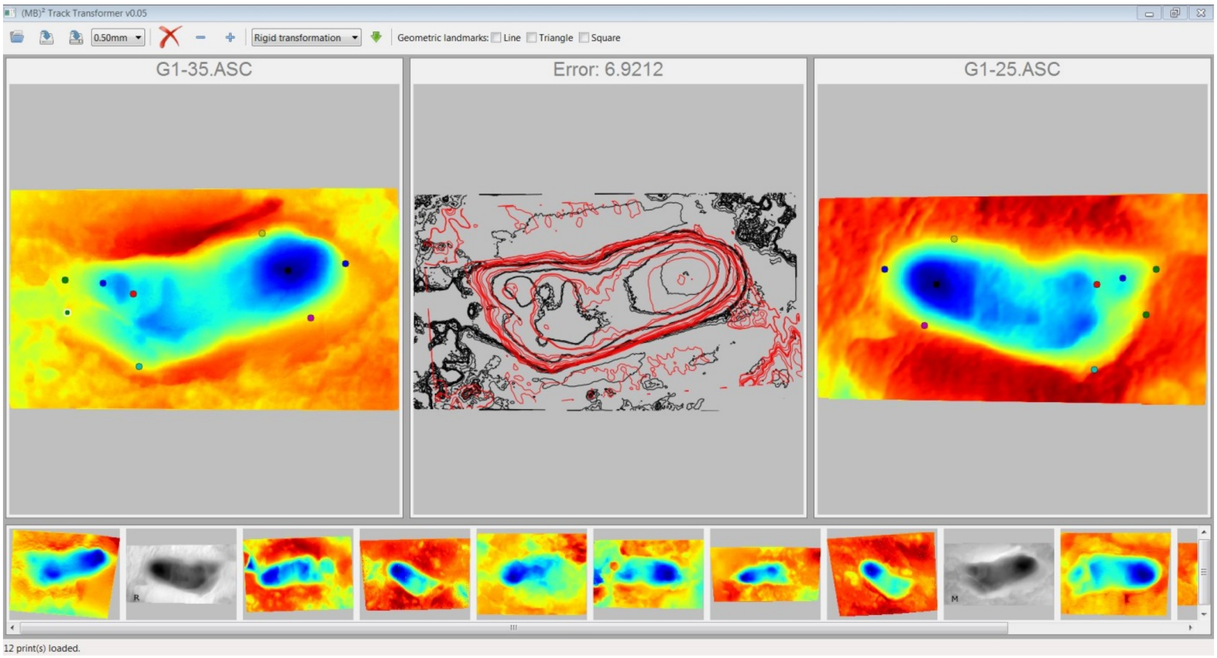

Figure S1: Track Transformer showing the registration of tracks from the G1-Trail at Laetoli. Note that the tracks do not need to be in the same orientation to be registered. The central panel shows the ‘live registration’ and the error denoting the Root Mean Squared Error averaged over all corresponding landmark pairs. The ‘master’ track is shown on the left hand side and in this case is G1-35. The data was captured using a Konica-Minolta Vi-900 scanner, processed in Foot Processor (http://footprints.bournemouth.ac.uk/).

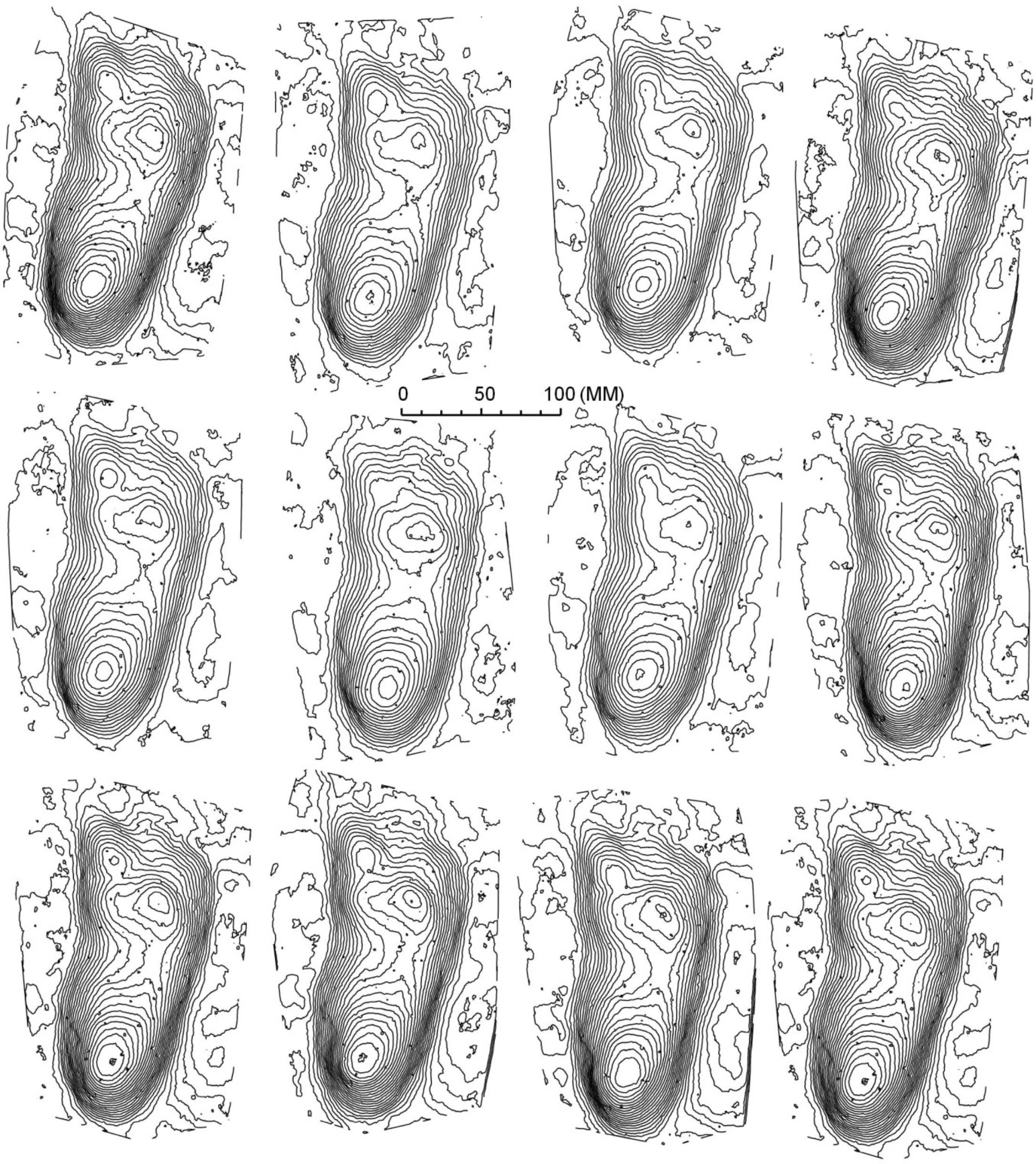

Figure S2: A series of nine contour maps for means of the G1-Trail generated by different operators. The variation between these is small and can be removed by creating a 'super' mean of each of the means. Contour interval is 1 mm. The mean is based on eleven individual tracks (G1-23, G1-25, G1-26, G1-27, G1-31, G1-33, G1-34, G1-35, G1-36, G1-37, G1-39). The operator variance is small but can also be filtered out by computing a 'super' mean from all of these registrations. The data was captured using a Konica-Minolta Vi-900 scanner, processed in Foot Processor (http://footprints.bournemouth.ac.uk/) and ArcGIS Version 10 (http://www.esri.com/software/arcgis).

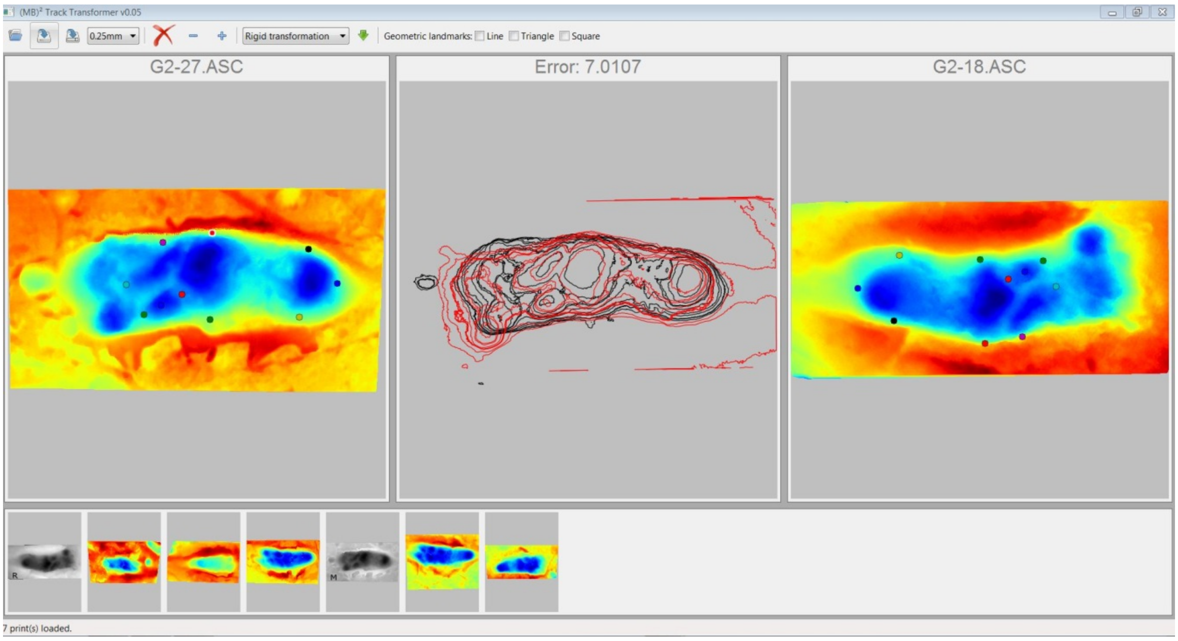

Figure S3: Example of registration based on the G3 tracks within selected tracks from the G2/G3 Trail. Registration was repeated multiple times and by different operators. The data was captured using a Konica-Minolta Vi-900 scanner, processed in Foot Processor (http://footprints.bournemouth.ac.uk/) and ArcGIS Version 10 (http://www.esri.com/software/arcgis).
